# Not All Cancer Survivors Respond to a 4-Week mHealth Exercise Fatigue Intervention: Who Are the Responders?

**DOI:** 10.3390/curroncol32120706

**Published:** 2025-12-15

**Authors:** Morgan Emmi, Myriam Filion, Yingwei Yao, Anna L. Schwartz, Diana J. Wilkie, Saunjoo L. Yoon

**Affiliations:** 1 Department of Biobehavioral Nursing Science, Center for Palliative Care Research and Education, College of Nursing, University of Florida, Gainesville, FL 32610, USA; mkemmi02@gmail.com (M.E.); y.yao@ufl.edu (Y.Y.); diwilkie@ufl.edu (D.J.W.); 2 Faculty of Kinesiology, Sport, and Recreation, College of Health Sciences, University of Alberta, Edmonton, AB T6G 2R3, Canada; mfilion@ualberta.ca; 3 Coleman Health LLC., Parks, AZ 86018, USA; annalschwartz@icloud.com

**Keywords:** cancer, fatigue, personalized intervention, responders, precision oncology, exercise, survivorship, response analysis

## Abstract

Cancer-related fatigue (CRF) is prevalent and onerous for cancer survivors. Not all survivors respond equally to interventions, but the characteristics distinguishing responders and non-responders are often unknown. This secondary analysis study compared baseline characteristics for responders (CRF reduction ≥2 points), non-responders, and those lost to follow-up using data from a two-group pre-test/post-test trial of a four-week exercise intervention compared to usual care. Included were 278 adult cancer survivors, with a mean age of 52.2 ± 11.9, 65% (180/278) female, and 90% (250/278) Caucasian. Of these, 77 (28%) were responders, 153 (55%) were non-responders, and 48 (17%) were lost to follow-up. At baseline, participants completed the 6-item Schwartz Cancer Fatigue Scale, with responses from 1 (not at all) to 5 (extremely fatigued) and a total score ranging 6–30. In the intervention group, 35% (49/141) reported decreased fatigue, 24% (34/141) reported increased fatigue, 25% (35/141) had minimal change, and 16% (23/141) were lost to follow-up. In the control group, 20% (28/137) reported decreased fatigue, 39% (53/137) reported increased fatigue, 23% (31/137) had minimal change, and 18% (25/137) were lost to follow-up. Responders in both groups reported higher baseline fatigue than non-responders, with mean differences of 5.2 (95% CI: 3.6–6.8) and 5.4 (95% CI: 3.4–7.3) for intervention and usual care, respectively. Higher baseline fatigue was found in responders compared to non-responders, regardless of group assignment, suggesting that those with a greater fatigue burden may have derived more benefit from exercise for CRF or a regression to the mean effect.

## Simple Summary

Cancer-related fatigue (CRF) is prevalent and onerous for cancer survivors. However, not all survivors respond equally to interventions. Identifying the different characteristics of responders and non-responders during a study is important to tailoring effective clinical interventions. Responder analysis findings of our exercise intervention trial indicated that responders (CRF reduction ≥2 points) and non-responders in both intervention and usual care groups mainly differed on baseline fatigue. Higher baseline fatigue levels (difference of 5 points) were observed in responders regardless of group assignment. However, the intervention group had a higher number of responders and significantly lower CRF at the end of the study. Findings suggest that those with a greater fatigue burden may have derived more benefit from exercise for CRF. Balancing study groups on baseline CRF levels is essential. CRF levels should be considered in designing effective personalized and precision exercise interventions to improve survivorship quality for all cancer survivors.

## 1. Introduction

Cancer-related fatigue (CRF) is the most frequent and debilitating symptom during and after cancer treatment and undermines survivorship outcomes [1,2,3], including functional capacity and quality of life throughout survivorship [4,5]. CRF is defined as a “distressing, persistent, subjective sense of physical, emotional, and/or cognitive [...] exhaustion related to cancer” [6] with prevalence ranging from 11% to 99% [7]. CRF goes beyond “normal” tiredness and weakness in that it is not relieved by rest or sleep, and occurs both as a side effect of cancer treatment as well as a manifestation of the cancer itself [8].

Pharmacologic treatments for CRF (e.g., psycho-stimulants, L-carnitine, and Modafinil) have shown limited effectiveness due to side effects and drug–drug interactions, especially considering the complex medication regimens and the management of cancer and cancer-related symptoms [9]. The National Comprehensive Cancer Network (NCCN) [10] and the American College of Sports Medicine [11] recommend exercise as a nonpharmacologic, evidence-based practice for its effectiveness to improve functional capacity and reduce CRF. However, exercise must be individualized to the survivor’s specific needs and abilities to have the most therapeutic effect and to prevent worsening fatigue [10].

Tailored interventions such as mobile health (mHealth) exercise education can promote knowledge about and reduce CRF, strengthen self-efficacy, and increase exercise [2,12]. An exercise mobile application (app) that provides an individualized exercise regimen to cancer survivors with CRF would also be instrumental in helping survivors, regardless of geographic location and personal barriers [13].

Few studies have focused on the inter-individual variability in response to exercise interventions for those living with cancer, with significant gaps remaining. A study to explore response to aerobic exercise by going beyond VO_2_ max alone, highlighted the importance of considering a wider range of physiological outcomes when characterizing who benefits from exercise [14]. Another responder analysis of lung cancer survivors undergoing preoperative home-based exercise training focused on quality of life outcomes and showed that not all participants experienced benefits despite overall group improvements [15]. Padilla et al. [16] examined how classifying individuals as responders or non-responders *without proper controls* can produce misleading results. A common flawed strategy has been to divide all participants based on their baseline outcome values and observe if those values show improvements. Although this technique suggests that those with more room to improve should show better results, it violates the assumption of statistical independence. The randomized controlled trial (RCT) of a mHealth intervention [2] showed an overall reduction in fatigue levels in cancer survivors. However, individual responses to the intervention varied, with some participants reporting substantial reductions in fatigue, whereas others described minimal to no change in fatigue levels [2], which raised questions about the variability in response to the intervention.

Results from prior studies demonstrate the importance of evaluating whether baseline symptoms, demographics, or clinical factors differ between responders and non-responders in exercise interventions for CRF. In this responder analysis, we examined the characteristics of responders (≥2 points pre–post reduction in CRF) and non-responders who participated in a previously published exercise intervention trial that showed statistically significant CRF reduction in the intervention group compared to the usual care group [2]. Addressing the research gap, our study applied responder analysis in both the experimental and usual care groups to inform the development of more personalized approaches to CRF management that support the survivorship needs of individuals with cancer.

## 2. Materials and Methods

### 2.1. Study Population and Design

This further analysis of an exercise RCT used data from a published two-group randomized controlled trial (RCT) of a four-week exercise education and prescription intervention, delivered via a digital tool, on CRF compared to a control group [2]. The exercise prescription, tailored to low and high fatigue levels, focused on walking four days per week with increased exercise duration over the four-week study period. There was much variability within groups, warranting additional responder analysis to inform future CRF studies for cancer survivors. The institutional review board approved the RCT. All participants consented to the use of their data for future research. The data were de-identified for this analysis.

**Figure 1:**
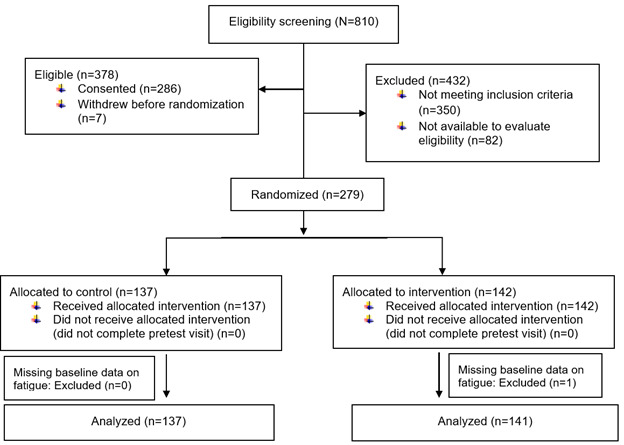
CONSORT diagram: Exercise for fatigue responder analysis study.

Cancer survivors were recruited from and completed the procedures at a university-affiliated cancer center. Inclusion criteria were adults 18 years or older who (a) had a diagnosis of cancer at any stage, (b) were receiving cancer therapy or continuing care at the study site, and (c) spoke and read English. Exclusion criteria were (a) physically unable to complete study questionnaires and study interventions (e.g., blindness, cognitive impairment), or (b) participating in other fatigue studies at the study site. As shown in Figure 1, one participant who was missing baseline data on fatigue was excluded from this analysis. Details of the study design, data collection, and description of the intervention are in the previous publication [2].

### 2.2. Measurements

**Fatigue**: The Schwartz Cancer Fatigue Scale (SCFS-6) was used to evaluate CRF with an interactive touch screen [17]. The SCFS-6 is a valid and reliable 6-item measure of CRF. Each item is scored from 1 (not at all) to 5 (extremely) about the feeling of CRF in the past 2–3 days. The total score is the sum of the six items, ranging from 6 to 30 [17,18]. A higher score indicates a higher level of CRF. The SCFS is sensitive to detecting changes in CRF level over time (*p* < 0.001) [19]. A study with a large effect size (Cohen’s *d* 0.695, 95% CI 0.235–0.975) found no significant differences in CRF between types of cancer [20]. The minimum clinically important difference (MCID) over time of the SCFS-6 is a decrease of 2.1 points [19]. Since SCFS-6 score changes are integers, we chose a priori a decrease of 2 (which is much closer to 2.1 than 3) as the cutoff for responders.

**Pain:** Pain intensity was measured using a tablet-based Pain Intensity Numerical Scale (PINS) [21], where participants rated their *current* pain intensity and pain intensity at its *least* and *worst* during the past 24 h. The PINS was rated on a scale from 0 (no pain) to 10 (pain as bad as it can be). The three scores were averaged to produce the average pain intensity. Any of the three scores missing would therefore result in a missing average pain intensity.

**Satisfaction:** Satisfaction with fatigue was assessed using a tablet-based question: “Are you satisfied with your fatigue level?” Participants responded by selecting *Yes*, *No*, or *Not sure.* As a single item, it is face valid. It has been used as a measure for satisfaction with symptom levels, such as pain level, with cognitive interview and focus group evidence of its validity in cancer and other populations.

**Demographic characteristics:** Data were collected via the tablet at the end of the pre-test visit, including self-reported information such as age, race, ethnicity, gender, marital status, education, and income. If participants were unable to complete all study measures during the pre-test visit, demographic questions were deferred and completed during the post-test visit.

### 2.3. Statistical Analysis

The analysis included responders, non-responders, and those lost to follow-up. Responders were defined as having ≥2 points of improvement in SCFS fatigue level at 4 weeks post-intervention compared to their pre-intervention level. Non-responders included participants whose fatigue remained unchanged or changed by ±1 point and those whose fatigue worsened by 2 points or more. Descriptive statistics were conducted to summarize demographic and baseline clinical characteristics by response categories within each group. Independent t tests and Fisher’s tests were used for pairwise comparison between responders and non-responders of each group on demographic and baseline clinical characteristics. For the baseline value of the primary outcome variable (fatigue), we also compared those lost to follow-up to responders and non-responders. When conducting these tests, we treated missing values of categorical variables as a separate category. For baseline pain levels, we excluded the 3 subjects missing pain data. We focused on comparisons within each group, as the groups were assigned different treatments, which would elicit different types of responses. Binary logistic regression analysis, including multiple predictors, was performed. We performed two regression analyses: (1) responders versus non-responders; (2) responders versus non-responders/lost to follow-up. The first approach utilized the complete-case analysis, assuming that participants were lost to follow-up at random, while the second approach, by grouping non-responders and those lost to follow-up, assumed that those lost to follow-up were non-responders. The reality most likely lay somewhere in between. The coefficient estimates of these two approaches were likely both biased, but together informed the values of true effects. A *p*-value less than 0.05 was considered statistically significant. For this exploratory analysis, we did not adjust for multiple testing.

## 3. Results

### 3.1. Sample Characteristics

A total of 278 cancer survivors with baseline fatigue scores were included (Table 1), including 48 (17%) participants who were lost to follow-up after the baseline measures due to progressive illness, relocation, or failure to return for follow-up. Table 1 presents the demographic characteristics, cancer stages, baseline fatigue, and baseline pain levels by intervention (n = 141) and control (n = 137) groups. Overall, the baseline mean and standard deviation (SD) for fatigue and pain levels were 14.3 ± 5.1 and 1.9 ± 1.9 in the intervention group, and 13.9 ± 5.0 and 2.7 ± 2.3 in the control group, respectively (Table 1).

### 3.2. Characteristics of Responders and Non-Responders

Of all participants combining the control and intervention groups, 28% (77/278) were responders and 55% (153/278) were non-responders. In the intervention group, 35% (49/141) were classified as responders, with reported improved fatigue levels of 2 points or higher; 49% (69/141) were non-responders; and 16% (23/141) were lost to follow-up (Table 2). In contrast, 20% (28/137) of the control group were classified as responders, 61% (84/137) were non-responders, and 18% (25/137) were lost to follow-up (Table 2).

As shown in Table 2, responders in the intervention and control groups reported mean baseline fatigue levels that were 5 points higher than non-responders (17.6 ± 4.6 vs. 12.4 ± 3.8 for intervention and 17.5 ± 4.4 vs. 12.2 ± 4.5 for control, *p* < 0.001 for both). The 95% CIs for the mean baseline fatigue difference between responders and non-responders were 3.6–6.8 for intervention and 3.4–7.3 for usual care. On the other hand, participants lost to follow-up reported mean baseline fatigue levels between those of non-responders and responders (13.4 ± 6.3, intervention; 15.7 ± 4.6, control). In the intervention group, the mean difference was −4.2 (*p* < 0.001; 95% CI [−6.5, −1.9]) between lost to follow-up and responders and 1.0 (*p* = 0.35; 95% CI [−1.1, 3.2]) between lost to follow-up and non-responders. In the control group, the mean difference was −1.8 (*p* = 0.14; 95% CI [−4.2, 0.6]) between lost to follow-up and responders and 3.6 (*p* = 0.001; 95% CI [1.6, 5.6]) between lost to follow-up and non-responders.

At baseline, a greater proportion of responders (64%; 49/77) reported being unsatisfied with their fatigue levels, compared to 35% (53/153) of non-responders. Conversely, 46% (71/153) of non-responders reported being satisfied with their fatigue levels, whereas only 10% (8/77) of responders did. The difference was statistically significant in both groups (*p* = 0.02 for control and *p* < 0.001 for intervention), with responders more likely to be dissatisfied with their fatigue levels than non-responders at baseline.

Relative to responders, the non-responders in the intervention group were more likely to be non-Caucasian (*p* = 0.02). The estimated odds ratio was 5.5 (95% CI [1.2, 25.4]). The responders and non-responders did not differ significantly on other characteristics. Those lost to follow-up in both groups had substantially more missing demographic data than the responders or non-responders (Table 2).

We performed binary logistic regression of responders versus non-responders/lost to follow-up (Table 3) and responders versus non-responders (Table 4), including as predictors the baseline fatigue levels, the baseline satisfaction with fatigue, and race for each group, separately. The baseline fatigue was consistently associated with being a responder. The negative association between being satisfied with baseline fatigue and being a responder was just statistically significant in this sample. The other predictors were not statistically significant when adjusting for multiple predictors.

### 3.3. Pre–Post Intervention Change in Fatigue by Study Group

**Figure 2:**
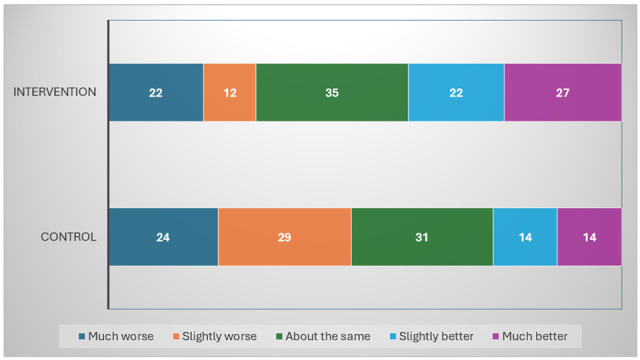
Pre–Post change in Schwartz Cancer Fatigue Scale by intervention and control group participants who completed the 4-week trial. The numbers reported are the exact counts of subjects in each category. Percentages are calculated exclusively from participants with complete pre- and post-intervention fatigue data. (N = 230: 118 intervention group; 112 control group; 48 missing; much worse = increase of 5 or more; slightly worse = increase of 2–4; about the same = magnitude of change 1 or 0; slight better = decrease of 2–4; much better = decrease of 5 or more.)

Figure 2 illustrates the distribution of change in fatigue scores, as measured by the SCFS, from pre- to post-intervention across the control and intervention groups. In this analysis, we focused exclusively on participants that have reported both baseline and post-intervention fatigue levels, including 118 in the intervention group and 112 in the control group. In the intervention group (n = 118), responders, defined as participants reporting a reduction in fatigue of 2 points or more, represented 42% (49/118) of the sample, including 23% (27/118) classified as much better (reduction of 5 points or more) and 19% (22/118) as slightly better (reduction of 2 to 4 points). In the control group (n = 112), 25% (28/112) of participants were classified as responders, reporting either a much better (13%; 14/112) or slightly better (13%; 14/112) level of fatigue.

Regarding non-responders, a similar proportion of participants in the control and intervention groups reported no change or a minimal change (magnitude ≤ 1) in fatigue, with 28% (31/112) and 30% (35/118), respectively. However, a greater proportion of participants in the control group (47%; 53/112) reported slightly worse (increase of 2 to 4 points) or much worse (increase of 5 points or more) fatigue than the intervention group (29%; 34/118).

## 4. Discussion

This further analysis of a RCT focused on the characteristics of the responders and non-responders to a mHealth exercise intervention for managing CRF. Responders were defined as participants with 2 points or more fatigue reduction from pre- to post-intervention. The one characteristic that consistently differed between responders and non-responders was the mean baseline CRF levels for the intervention and control groups, which remained highly significant after adjusting for other variables. Responders reported a higher baseline CRF (5 points) than the non-responders. Responders were more likely to be unsatisfied with their fatigue levels. None of the other demographic (age, sex, education level, marital status, and family income) or clinical (cancer stage, or pain intensity) characteristics differentiated the responders and non-responders. Notably, the participants with stage 4 cancers (43%) responded to the intervention similarly to their counterparts (with stages 1–3), suggesting their willingness to exercise to improve fatigue despite their advanced stage of cancer. The participants who were lost to follow-up reported a mean baseline CRF similar to that of responders and non-responders. Additionally, a higher proportion of those lost to follow-up had missing data.

Responders reported higher fatigue levels at the study baseline. This result suggests that individuals with a greater baseline fatigue burden may have higher motivation, greater capacity for improvement, or be more sensitive to mHealth exercise interventions. These individuals may be more motivated or responsive to improve their fatigue when exposed to mHealth exercise intervention. Other researchers found that survivors with advanced lung cancer demonstrated significantly improved CRF than those in the control group based on the findings of a meta-analysis (SMD = 0.33; 95% CI: −0.54 to −0.12; *p* = 0.00; I^2^ = 0.00%) [22]. Our finding that responders with higher fatigue levels showed greater improvement after only four weeks of intervention supports the hypothesis that greater fatigue burden may be associated with a faster or more pronounced response to exercise education. Conversely, it may take longer for participants with moderate fatigue to experience noticeable benefits, particularly from a low-to-moderate intensity intervention. Previous dose-finding studies in exercise oncology have shown a dose–response relationship between exercise intensity and fatigue reduction in an 8-week intervention [23]. For instance, a walking program of similar intensity did not yield significant reductions in fatigue until after four weeks [24]. These study results suggest that the short duration of this intervention may limit the effects of exercise on fatigue in participants with a more moderate fatigue burden. Another possible explanation for our findings is regression to the mean.

A larger proportion of responders reported low baseline satisfaction with fatigue. Adjusting for baseline CRF, satisfaction with fatigue level at baseline was associated with responding in the intervention group but not in the control group, suggesting that dissatisfaction with fatigue may have motivated engagement with supportive resources or receptivity to behavioral change advocated by the intervention. Perceived need or readiness for change, as reflected in fatigue dissatisfaction, may influence who benefits most from mHealth exercise interventions. Other researchers found that cancer survivors with lower self-efficacy reported higher levels of fatigue, suggesting that interventions targeting self-efficacy may be particularly valuable for this group [25]. Another study [26] showed that an 8-week multilateral training program reduces perceived fatigue in cancer survivors. These findings also suggest that mHealth strategies may be particularly beneficial for individuals with lower self-efficacy, as they offer structure and guidance for managing symptoms and bolstering self-efficacy. As shown in a systematic review [27], mHealth may improve fatigue and self-efficacy in cancer survivors. Although we did not measure self-efficacy in an RCT, it may be an important variable for future research.

Differences in baseline pain levels were not statistically significant between responders and non-responders in this sample. However, it could still influence participants’ expectations and perceived need for support. Demographic variables such as age, sex, and cancer stage also did not significantly distinguish responders from non-responders. Similarly to our findings, Pimenta et al. reported no significant differences in baseline demographic and clinical characteristics between responders and non-responders [15]. This lack of differentiation underscores the importance of moving beyond simple socio-demographic factors when attempting to predict responsiveness to mHealth interventions to reduce fatigue. Other influential variables may include self-efficacy, symptom burden, treatment history, comorbidities, or prior exercise behavior [25]. As the need for precision medicine continues to grow, these findings reinforce the importance of designing tailored interventions that reflect individual profiles and symptom experiences rather than relying solely on generalized characteristics.

Although these findings are encouraging, this study has some limitations that should be considered. The observed trend could reflect a regression to the mean effect, whereby participants with extreme baseline values naturally experience a return toward average values over time [28]. Concern about regression to the mean is mitigated somewhat by the randomized controlled design since both the control and experimental groups had extreme CRF scores and regression to the mean would occur in both groups. Also, other study design features reported previously include a few restrictions on inclusion and exclusion criteria to reflect the real-world population, similar attrition in both groups to reduce attrition bias, and attention to clinician and researcher blinding to group assignment. The Hawthorne effect (performance bias), however, may have been a residual source of bias. The control group’s higher prevalence of worsening fatigue strengthens the argument that the intervention, even when delivered through mHealth and over a short time, may have provided a protective effect for participants with initially high CRF levels. This possibility aligns with a robust body of evidence supporting the effectiveness of exercise in reducing CRF [11,29]. Missing data from participants lost to follow-up also limits the strength of conclusions about non-responders. Disengagement may have been related to symptom burden, lack of motivation, or other life circumstances not captured in this study.

Our secondary analysis included a large and diverse sample, capturing both intervention and control group responders. The inclusion of responders in the control group enhances the ecological validity of our findings, reflecting real-world conditions where individuals may benefit from minimal engagement. Another strength of this study is the clear and evidence-based definition of the “response threshold,” grounded in the established MICD for improvement in CRF. The SCFS, used to measure fatigue in this study, is reliable and valid, with an MICD of 2.1 points. It ensures that observed changes in fatigue are clinically meaningful.

Findings have implications for clinical practice and the design of future studies. Since participants reported dissatisfaction with even low fatigue levels, clinicians and researchers should consider all levels of fatigue as a serious health issue and provide interventions tailored to the specific fatigue level. We stratified fatigue levels as high (SCFS score ≥15) or low (SCFS score ≤14); these values can serve as cut points for balancing groups and interventions targeted to fatigue levels. The strategy of gradually increasing exercise duration and intensity for patients with high fatigue levels is a key implication for practice.

These results provide a foundation for future work that incorporates more complex variables to better distinguish between responders and non-responders and inform the design of targeted, scalable interventions that reflect the principles of precision oncology care across the cancer survivorship continuum. Tailored exercise interventions and balancing groups by low and high fatigue levels is a critical implication for randomized research designs.

## 5. Conclusions

Overall, this study suggests that baseline fatigue severity may predict responsiveness to exercise interventions for CRF. First, Cancer survivors with higher initial fatigue levels were more likely to benefit from the mHealth intervention, indicating a potential path toward personalized exercise support delivered through digital platforms. Secondly, dissatisfaction with fatigue may indicate a greater readiness or need for intervention. Finally, the finding that even some control group participants demonstrated improvement speaks to the broader behavioral impact of mHealth research engagement and the need for study samples of sufficient size to detect the real world intervention effectiveness.

## Figures and Tables

**Table 1:** Baseline characteristics of the main study population by intervention and control groups (N = 278).

		Intervention (n = 141)	Control (n = 137)
**Age, Mean (SD),** [95% CI]		52.2 (11.9) [50.2, 54.2]	52.3 (13.2) [50.1, 54.6]
**Baseline Fatigue ^#^, Mean (SD),** [95% CI]		14.3 (5.1) [13.5, 15.2]	13.9 (5.0) [13.1, 14.8]
Baseline Pain *, Mean (SD), [95% CI]		1.9 (1.9) [1.6, 2.2]	2.7 (2.3) [2.3, 3.1]
**Gender, n (%)**	Female	91 (65%)	89 (65%)
	Male	50 (35%)	48 (35%)
**Marital status**, **n (%)**	Married/Partnered	73 (52%)	79 (58%)
	Single	38 (27%)	35 (26%)
	Widowed	8 (6%)	3 (2%)
	Missing	22 (16%)	20 (15%)
**Education**, **n (%)**	High school or less	29 (21%)	33 (24%)
	Some college	39 (28%)	44 (32%)
	College or higher	62 (44%)	49 (36%)
	Missing	11 (8%)	11 (8%)
**Annual Family Income**, **n (%)**	<$10,000	8 (6%)	12 (9%)
	$11–20,000	12 (9%)	9 (7%)
	$21–30,000	11 (8%)	11 (8%)
	$31–40,000	8 (6%)	9 (7%)
	$41–50,000	21 (15%)	11 (8%)
	>$50,000	60 (43%)	50 (36%)
	Missing	21 (15%)	35 (26%)
**Race**, **n (%)**	Caucasian	123 (87%)	127 (93%)
	Other	18 (13%)	10 (7%)
**Cancer Stage, n (%)**	1–3	80 (57%)	74 (54%)
	4	60 (43%)	54 (39%)
	Missing	1 (1%)	9 (7%)

^#^ Observed baseline fatigue scores ranged from 6–27 for the usual care and 6–26 for intervention groups; * Variable was missing for 3 participants in the control group; Abbreviations: SD, standard deviation.

**Table 2:** Baseline characteristics of the study population in the RCT by responders, non-responders, and lost to follow-up. Mean (SD) and 95% CI are shown for continuous variables, and counts (%) are shown for categorical variables.

	Intervention (n = 141)			Control (n = 137)		
	Non-Responder	Responder	Lost to Follow Up	Non-Responder	Responder	Lost to Follow Up
	n = 69 (49%)	n = 49 (35%)	n = 23 (16%)	n = 84 (61%)	n = 28 (20%)	n = 25 (18%)
**Age**	52.2 (12.9) [49.1, 55.3]	51.5 (11.4) [48.2, 54.8]	53.7 (9.7) [49.5, 57.9]	53.6 (13.3) [50.7, 56.5]	49.2 (12.8) [44.3, 54.2]	51.4 (13.1) [46.0, 56.7]
**Fatigue Level**	12.4 (3.8) [11.5, 13.3]	17.6 (4.6) [16.3, 18.9]	13.4 (6.3) [10.7, 16.1]	12.2 (4.5) [11.2 13.1]	17.5 (4.4) [15.8, 19.2]	15.7 (4.6) [13.8, 17.6]
Pain Level *	1.7 (1.7) [1.2, 2.1]	2.2 (2.1) [1.6, 2.8]	1.9 (1.9) [1.0, 2.7]	2.3 (2.2) [1.8, 2.8]	3.1 (2.3) [2.2, 4.0]	3.5 (2.6) [2.4, 4.6]
**Satisfied with Fatigue Level, n (%)**						
No	22 (32%)	33 (67%)	9 (39%)	31 (37%)	16 (57%)	15 (60%)
Not sure	11 (16%)	11 (22%)	5 (22%)	16 (19%)	8 (29%)	6 (24%)
Yes	35 (51%)	4 (8%)	9 (39%)	36 (43%)	4 (14%)	4 (16%)
Missing	1 (1%)	1 (2%)	0 (0%)	1 (1%)	0 (0%)	0 (0%)
**Gender, n (%)**						
Female	39 (57%)	35 (71%)	17 (74%)	56 (67%)	16 (57%)	17 (68%)
Male	30 (43%)	14 (29%)	6 (26%)	28 (33%)	12 (43%)	8 (32%)
**Marital Status, n (%)**						
Married/Partnered	33 (48%)	29 (59%)	11 (48%)	52 (62%)	20 (71%)	7 (28%)
Single	18 (26%)	16 (33%)	4 (17%)	24 (29%)	6 (21%)	5 (20%)
Widowed	7 (10%)	1 (2%)	0 (0%)	1 (1%)	1 (4%)	1 (4%)
Missing	11 (16%)	3 (6%)	8 (35%)	7 (8%)	1 (4%)	12 (48%)
**Education, n (%)**						
High school or less	16 (23%)	9 (18%)	4 (17%)	20 (24%)	8 (29%)	5 (20%)
Some college	17 (25%)	17 (35%)	5 (22%)	27 (32%)	10 (36%)	7 (28%)
College or higher	33 (48%)	23 (47%)	6 (26%)	36 (43%)	10 (36%)	3 (12%)
Missing	3 (4%)	0 (0%)	8 (35%)	1 (1%)	0 (0%)	10 (40%)
**Annual Family Income, n (%)**						
<$10,000	4 (6%)	4 (8%)	0 (0%)	7 (8%)	4 (14%)	1 (4%)
$11–20,000	5 (7%)	5 (10%)	2 (9%)	5 (6%)	2 (7%)	2 (8%)
$21–30,000	6 (9%)	3 (6%)	2 (9%)	6 (7%)	3 (11%)	2 (8%)
$31–40,000	7 (10%)	1 (2%)	0 (0%)	6 (7%)	3 (11%)	0 (0%)
$41–50,000	14 (20%)	5 (10%)	2 (9%)	7 (8%)	2 (7%)	2 (8%)
>$50,000	25 (36%)	26 (53%)	9 (39%)	36 (43%)	8 (29%)	6 (24%)
Missing	8 (12%)	5 (10%)	8 (35%)	17 (20%)	6 (21%)	12 (48%)
**Race, n (%)**						
Caucasian	56 (81%)	47 (96%)	20 (87%)	79 (94%)	25 (89%)	23 (92%)
Other	13 (19%)	2 (4%)	3 (13%)	5 (6%)	3 (11%)	2 (8%)
**Cancer Stage, n (%)**						
1–3	42 (61%)	27 (57%)	10 (43%)	46 (55%)	14 (50%)	14 (56%)
4	27 (39%)	21 (43%)	12 (52%)	35 (42%)	13 (46%)	6 (24%)
Missing	0 (0%)	0 (0%)	1 (4%)	3 (4%)	1 (4%)	5 (20%)

* Variable missing for 3 participants in the control group, including 1 responder and 2 non-responders; responders = posttest fatigue reduction of 2 or higher; Abbreviations: SD, standard deviation.

**Table 3:** Regression analysis of responder versus non-responder/lost to follow-up.

Group	Predictor	Estimate	Std Err	OR [95% CI]	*p*
Intervention	Baseline fatigue	0.176	0.051	1.19 [1.08, 1.32]	**<0.001**
	Baseline Satisfaction = Not sure	0.029	0.512	1.03 [0.38, 2.81]	0.96
	Baseline Satisfaction = Yes	−1.353	0.652	0.26 [0.07, 0.93]	**0.04**
	Race = White	1.306	0.875	3.69 [0.66, 20.5]	0.14
Control	Baseline fatigue	0.208	0.060	1.23 [1.09, 1.38]	**0.001**
	Baseline Satisfaction = Not sure	0.759	0.584	2.14 [0.68, 6.72]	0.19
	Baseline Satisfaction = Yes	0.036	0.740	1.04 [0.24, 4.42]	0.96
	Race = White	−0.679	0.845	0.51 [0.10, 2.66]	0.42

Bold indicates statistically significant values.

**Table 4:** Regression analysis of responder versus non-responder.

Group	Predictor	Estimate	Std Err	95% CI	*p*
Intervention	Baseline fatigue	0.229	0.064	1.26 [1.11, 1.42]	**<0.001**
	Baseline Satisfaction = Not sure	0.036	0.571	1.04 [0.34, 3.17]	0.95
	Baseline Satisfaction = Yes	−1.329	0.677	0.26 [0.07, 1.00]	**0.05**
	Race = White	1.554	0.921	4.73 [0.78, 28.8]	0.09
Control	Baseline fatigue	0.244	0.065	1.28 [1.12, 1.45]	**<0.001**
	Baseline Satisfaction = Not sure	0.746	0.631	2.11 [0.61, 7.26]	0.24
	Satisfaction = Yes	−0.051	0.750	0.95 [0.22, 4.13]	0.95
	Race = White	−0.513	0.924	0.60 [0.10, 3.66]	0.58

Bold indicates statistically significant values.

## Data Availability

The data analyzed during the current study are available from the corresponding author upon reasonable request.
